# Understanding COVID-19: From Dysregulated Immunity to Vaccination Status Quo

**DOI:** 10.3389/fimmu.2021.765349

**Published:** 2021-11-11

**Authors:** Ruby A. Escobedo, Dhiraj K. Singh, Deepak Kaushal

**Affiliations:** ^1^ Southwest National Primate Research Center, Texas Biomedical Research Institute, San Antonio, TX, United States; ^2^ The Integrated Biomedical Sciences (IBMS) Graduate Program, University of Texas Health Sciences Center at San Antonio, San Antonio, TX, United States

**Keywords:** COVID-19, 2019-nCoV, SARS-CoV-2, Coronavirus, beta-coronavirus, vaccine development, animal models, immune responses

## Abstract

The development of vaccines against infectious diseases has helped us battle the greatest threat to public health. With the emergence of novel viruses, targeted immunotherapeutics ranging from informed vaccine development to personalized medicine may be the very thing that separates us between life and death. Late in 2019, severe acute respiratory syndrome coronavirus 2 (SARS-CoV-2), the etiological agent of coronavirus disease 2019 (COVID-19), made a remarkable entrance to human civilization, being one of many to cross the species barrier. This review discusses the important aspects of COVID-19, providing a brief overview of our current understanding of dysregulated immune responses developed using various experimental models, a brief outline of experimental models of COVID-19 and more importantly, the rapid development of vaccines against COVID-19.

## Introduction

The threat to human health posed by a pathological agent has long been a global public health concern for as long as we can remember. According to the Centers for Disease Control and Prevention, there are six major factors that contribute to disease emergence and re-emergence, which include changes in human demographics and behavior, advances in technology and changes in industry practices, economic development and changes in land-use patterns, dramatic increases in volume and speed of international travel and commerce, microbial adaptation and change (one of the more challenging factors), and the breakdown of public health capacity required for infectious diseases at the local, state, national, and global levels (1).

A more common theme of emerging pathogens is that the majority are of animal origin and have been viral. The first global pandemic of the twenty-first century was attributed to the emergence of severe acute respiratory syndrome coronavirus (SARS-CoV) in 2002 making its first appearance in Guangdong Province China. Nearly a decade later, Middle East respiratory syndrome coronavirus (MERS-CoV) emerged in 2012 in Saudi Arabia, and less than a decade later, another novel CoV emerged in Wuhan China late 2019. This novel CoV was later named severe acute respiratory syndrome coronavirus 2 (SARS-CoV-2) for its genetic and disease similarities to SARS-CoV and is the etiological agent of coronavirus disease 2019 (COVID-19).

SARS-CoV-2 is now considered a global pandemic and is a substantial threat to human health, with over 182 million confirmed cases and approximately 4 million deaths recorded in 215 countries and territories by July 2021 (2). While the pathology and immune responses of COVID-19 are similar to SARS, COVID-19 patients have varied clinical manifestations in which they will either remain asymptomatic or experience mild, moderate to severe symptoms (3). The unique variability of disease manifestations of COVID-19 has led to the development of several treatment and vaccine platforms in efforts to eradicate the disease (4–8).

In this review, we cover the more important aspects of what is currently known about SARS-CoV-2 based on unprecedented efforts from researchers and medical workers worldwide. We also provide a detailed review of how characterizing immune responses, notably dysregulated immune responses, to infection have aided the rapid development of vaccines and therapeutics for combating COVID-19. More importantly, we also discuss all known animal models used to study immunological responses and test novel treatments and vaccines against SARS-CoV-2.

## Immune Responses

The immune system is an extremely complex network of cells and signaling cascades, elegantly orchestrating the necessary responses to successfully eliminate the threat. This complex network involves subset interactions spanning from innate to adaptive immune responses (9, 10). As we learn to live with the newly emerging CoV, understanding how the host immune system responds to the virus and how the virus evolves to subvert immune recognition is of key importance for the development of efficient vaccines and medications for both prevention and treatment, particularly with long term efficacy. Many of the early inferences formulated for SARS-CoV-2 were on the parallel basis of the pathological and immunological features of other CoVs that target the lower respiratory tract (e.g. SARS-CoV-1 and MERS-CoV). Humoral and cell-mediated immune responses to SARS-CoV-2 were predicted from the first novel coronaviruses that emerged early in the century, SARS-CoV-1 and MERS-CoV, as well as other coronaviruses that cause the common cold. Because SARS-CoV-2 is similar to SARS-CoV-1, the instigated immune responses were thought to behave in a similar aspect.

Previous studies focusing on other CoV’s along with recent reports on SARS-CoV-2 encompassing preclinical animal models to clinical observations in human cohorts have allowed us to predict and define the cells that orchestrate the immune responses responsible for both protection and pathology (11–13). One of the key aspects for understanding why many patients develop different forms of the disease may be related to varied immune responses, specifically in the context of associated comorbidities. While targeting adaptive immune responses plays a critical role in achieving protection through vaccination, targeting innate immune responses can aid in combating immunopathological manifestations of COVID-19.

### Innate Immunity

**Figure 1:**
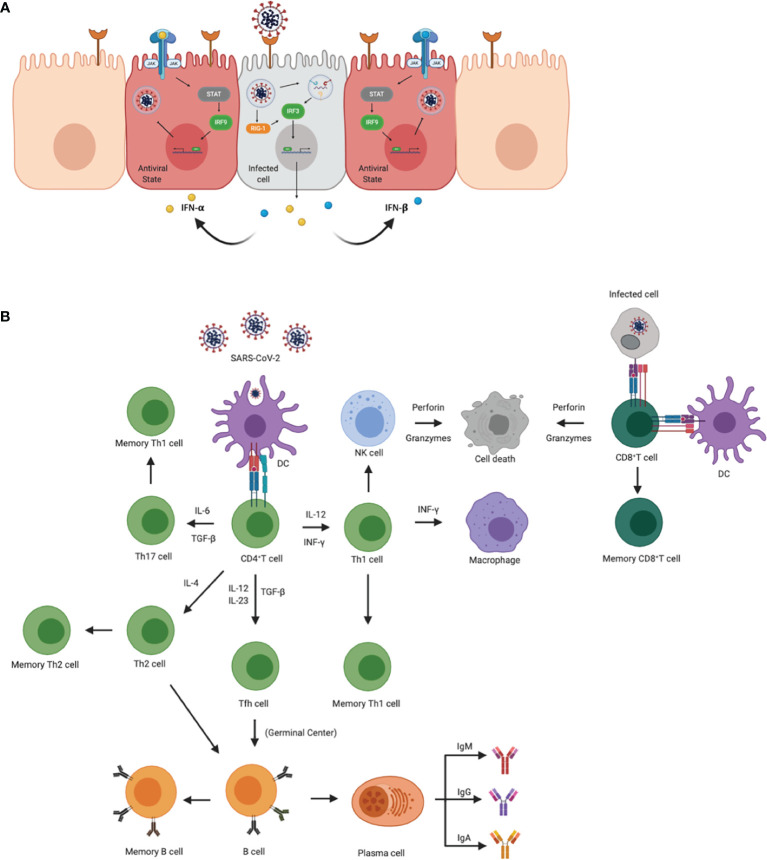
Innate and adaptive immune responses to SARS-CoV-2. **(A)** Cellular innate immune responses after SARS-CoV-2 enters the host cell through ACE2 receptors. Downstream signaling activates IRF1 and IRF3 to transcribe IFN-γ and IFN- α/β, respectively, leading to an antiviral state across neighboring cells. Once in the antiviral state, the cell is able to prevent the virus from propagating. **(B)** Adaptive immune activation after antigen presentation *via* APCs or infected cells. APCs, such as dendritic cells present viral antigens to CD4+ and CD8+ T cells. Activation of CD4+ T cells results in the clonal expansion of Th1, Th2, Th17 cells, and T follicular helper cells (Tfh). Th1 and Th17 cells attract macrophages and natural killer cells to the site of infection. Th2 and Tfh cells help in the activation of B cells in germinal centers, as well as the production of antibodies specific for viral antigens. CD8+ T cells are activated *via* APCs and/or infected cells. Once activated, CD8+ T cells transition to cytotoxic lymphocyte phenotypes and induce apoptosis in infected cells.

Like SARS and MERS, 3%- 20% of severe COVID-19 patients often progress to acute respiratory distress syndrome (ARDS), which is associated with an upregulation of proinflammatory cytokines and chemokines, implying an induction of a cytokine storm (14). A cytokine storm is mostly due to the de-regulated innate and adaptive immune responses, leading to an increase in circulating cytokines with a local origin and systematic circulation (**Figure 1A**). Lack of negative feedback mechanisms leads to dysregulated cytokine responses causing collateral damage in tissues (14, 15). The upregulation of such cytokines and chemokines are interleukin-1β (IL-1β), IL-8, IL-6, CXC-chemokine ligand 10 (CXCL10), and CC-chemokine ligand 2 (CCL2) (16). Other studies have demonstrated an increase in plasma levels of cytokines in intensive care unit COVID-19 patients such as IL-6, IL-2, IL-7, IL-10, granulocyte-colony stimulating factor, interferon-γ(IFN-γ), monocyte chemoattractant protein, macrophage inflammatory protein 1 alpha, and TNF-α (17). In SARS-CoV-2 acutely infected rhesus macaques, IFN-α, IL-1Ra, and IL-6, key components of a cytokine storm, were significantly elevated in bronchoalveolar lavage (BAL) fluid after 3 days post-infection, but normalized thereafter (18, 19). IFN-α levels were also elevated in plasma but not the other cytokines, suggesting localized early cytokine storm formation in the lungs. In addition to immune responses, cellular stress responses such as cellular senescence and programmed cell death including pyroptisis, apoptosis, and necroptosis may also influence the induction of cytokine deregulation (20). SARS-CoV-2 has been shown to trigger a variety of cell deaths, pyroptisis being the main cell death observed in the elderly population (21). Inflammation from cell death further enhances inflammatory responses through the release of PAMPs and DAMPs (**Figure 1A**) (21). COVID-19 patients that progress to ARDS is ultimately marked by the induction of these pro-inflammatory responses that lead to a cytokine storm (14, 22, 23). Therefore, understanding the molecular mechanisms that regulate virulence, pathogenesis, and disease outcomes is important.

SARS-CoV-2 is associated with an influx of specific myeloid cells to the lung, mostly macrophages, neutrophils, and plasmacytoid Dendritic cells (pDCs) and these subsets have also been observed to harbor viral antigens in the early phase of infection in a rhesus macaque model (18, 19). While this can be attributed to phagocytic uptake, the virus has also been shown to be capable of infecting tissue-resident alveolar macrophages and monocyte-derived alveolar macrophages (**Figure 1B**). Previous studies found that SARS-CoV can infect DCs altering their maturation, further raising the question of whether SARS-CoV-2 can impair DC function as well. Additionally, the absence of a marked classical Dendritic cell (cDCs) response during a SARS-CoV-2 infection can be attributed to independent studies showing marked viral antagonism of STAT1 phosphorylation, thus subverting antiviral IFN signaling (24, 25).

The initial immune responses are activated through pattern recognition receptors (PRR) mostly from antigen-presenting cells (APCs) that distinguish self from non-self-entities by binding to pathogen-associated molecular patterns (PAMPS). The key PRRs that play an important role in detecting coronaviruses are Toll-like receptor 7 (TLR-7) and TLR-8, as well as RIG-1, MDA-5. TLR-7 and TLR-8 are activated by single-stranded RNA (ssRNA) in endosomes, and RIG-1 and MDA-5 recognize cytosolic viral double-stranded RNA (dsRNA) that contain a 5’- triphosphate group, and/or lack a 5’ methyl cap (17, 26) (**Figure 1B**). One of the first responders to a viral infection are pDCs, which are critical for antiviral immune responses that generate the first wave of IFN-α; however, other host cells also express PRRs and generate proinflammatory cytokines and chemokines (27). Receptors that detect ssRNA, including TLR-7 and TLR-8, activate MyD88 which recruits adapter proteins to transduce signals to the downstream kinase complexes. The goal of these downstream signaling cascades is to activate IFN regulatory factor 3 (IRF3) and NFkB that regulate type 1 IFN, including IFN-α and -β. IFN will activate the signal transducer and activator of transcription (STAT) by the *Janus* family of tyrosine kinase (JAK) and activate transcription factors that aid in the expression of IFN-stimulated genes (ISGs) (28). This cascade of signals also occurs in host infected cells that secrete type 1 IFNs to surrounding cells and establish an antiviral state that helps limit viral replication and spread (**Figure 1A**).

While neighboring cells are entering an antiviral state, the inflammatory cytokines and chemokines attract myeloid cells, including macrophages, dendritic cells (DC) and neutrophils, as well as natural killer (NK) cells (**Figure 1B**). The combination of cells contributes to the activation of the adaptive immune response and induce a Type 1 T cell polarization. Through the induction of IFN-γ, NK cells can also activate leukocytes that activate antiviral responses and further induce proinflammatory cytokines. The activation, coordination and regulation of antiviral responses are almost entirely mediated by cytokine production. While these responses are important for the elimination of the virus, if they are not regulated, the sustained upregulation of proinflammatory cytokines and chemokines continues to recruit inflammatory monocytes to the site of infection which may induce tissue pathology. Additionally, COVID-19 demonstrated an increase in CXCL9 and CXCL16, chemoattractant of T or NK cells respectively; CCL8 and CCL2, recruiting monocytes and/or macrophages; and CXCL8, a neutrophil chemoattractant (29). Recruitment of these cells may be associated with the signature pathology in COVID-19 patients. Furthermore, inflammation-associated lung damage seen in severe patients may also be attributed to neutrophil extracellular traps (30), which were found in the airway compartment and neutrophil-rich inflammatory areas of the interstitium (31). Reactive oxygen species (ROS) have also been shown to increase the formation of NETs and suppress adaptive immune responses (32). These results suggest that NETs may be related to severe pulmonary complications of COVID-19.

#### Evasion of Innate Immunity

The IFN system has been recognized as a crucial frontline defender against viral infections (33). These cytokines are produced to modulate innate and adaptive immune responses which include dendritic cell maturation and the increase of macrophage phagocytosis, thus limiting the spread of the virus (34). However, viruses have evolved to avoid immune surveillance, thus allowing them to persist. CoVs, specifically, have evolved to avoid/antagonize the IFN system using three different mechanisms: 1) avoidance by physically shielding itself from the host, either through the production of proteins to methylate their own mRNA 5’ cap structures to avoid cytolytic PRRs, or using double-membrane vesicles (DMVs) to shield from cytolytic PRRs through physical avoidance 2) antagonizing the IFN system through non-structural proteins to prevent the induction of IFN, for example, nsp1 degrades mRNA including IFN messengers which impairs a proper immune response, and 3) antagonizing the IFN system to suppress signaling, thus delaying the antiviral state within the infected cell (35, 36). These evasion mechanisms can lead to a delayed IFN production as well as T and B cell priming- effectively leading to a slowed immune response for clearance and an increase in viral production. The avoidance/antagonizing characteristics against the IFN system are further supported by the low levels of IFN-I and IFN-III found in COVID-19 patients (29). Another study demonstrated overt but delayed type-I IFNs, and found many nonstructural and structural viral proteins that target host innate immune responses. In this study, they found that ORF6 specifically perturbs signaling pathways both upstream and downstream of IFN production, potentiating the extensive IFN production seen in COVID-19 patients (37). This conclusion is also supported by a study using reverse genetics to identify ORF3 and ORF6 to be major contributors of viral pathogenesis (38). Interestingly, another study found that SARS-CoV-2 membrane protein inhibits the production of type I and III IFNs induced by the cytosolic dsRNA-sensing pathway which is mediated by RIG-I/MDA-5–MAVS signaling. This study gives additional mechanistic insights into how SARS-CoV-2 can hamper innate immune responses (39). Extensive work performed in the NHP model of SARS-CoV-2 infection indicates that these animals mount robust, early IFN responses, and simultaneously clear the infection (18, 19, 40). On the other hand, many humans with late to end-stage COVID-19 are characterized by a “cytokine storm” with IFN as the key cytokine. Late to end-stage COVID-19 is not observed in NHPs (19). Therefore, further comparative research on early versus late IFN responses in NHPs and human beings is necessary to identify if IFN responses play a critical role in the control of SARS-CoV-2 infection or its progression. Elegant reagents to perform such experiments are available for NHPs (41).

In addition to interfering with the IFN system, SARS-CoV-2 has also been shown to infect antigen-presenting cells (APCs) including dendritic cells, macrophages and monocytes, disrupting their primitive function of activating adaptive immunity (24, 25, 40, 42). While these cells did not support viral production, their attenuated functions averted strong antibody but weak CD8 T cell responses (24, 25). Moreover, ORF8 encoded by SARS-CoV-2 has been shown to interact with MHC-I molecules in the ER and significantly downregulate their surface expression in various cell types (43). It has been postulated that MHC-I molecules are targeted for lysosomal degradation through an autophagy-dependent mechanism, and as consequence cytotoxic T cells inefficiently eliminate ORF8-expressing cells.

### Adaptive Immunity

Activated cells with myeloid lineages effectively present viral antigens through MHC/HLA molecules to stimulate the activation of T cells and B cells. Further activation elicits antigen-specific responses that lead to the development of effector T cells and the production of antibodies (**Figure 1B**) (44). After antigen presentation, naïve CD4+ and CD8+ T cells are activated and mature into T helper cells or cytotoxic lymphocytes (CTL), respectively (**Figure 1B**) (44). Upon activation, CTL cells help in clearing infection by inducing apoptosis through the release of perforin and granzymes, as well as recruit innate cells such as macrophages and NK cells (44) (**Figure 1B**). While the spike protein is responsible for receptor binding and membrane fusion of the virus, it also acts as a major antigen for humoral and cell-mediated immune responses being dominant over other viral antigens (**Figure 1B**) (45). Antibody production in viral infections may help neutralize the pathogen before gaining entry into the host cell. Humoral immunity in COVID-19 patients has shown to be short-lived and most SARS-CoV-2 antibodies show limited somatic hypermutation, thus lacking durability (45). This phenomenon may be attributed to the absence of germinal centers in the lymph node and spleen and Bcl-6-expression B cells, defective Bcl-6+ T follicular helper cell generation and differentiation, as well as dysregulated SARS-CoV-2 specific humoral immunity, found only in severely infected patients (45). Furthermore, additional studies are needed to determine the longevity and duration of SARS-CoV-2 specific antibodies.

Because continued T cell depletion is a hallmark of COVID-19, further elucidating immunological mechanisms of T cell responses may enhance immune reconstitution (marked by an increase in T cell numbers) after treatment. Two weeks after the onset of symptoms, SARS-CoV-2 specific CD4+ and CD8+ T cells are evident in the peripheral blood (PB) of COVID-19 patients. Additionally, PB CD4+ T cells expressed central memory phenotypes that dominantly produced Th1 cytokines and CD8+ T cells had a more effector memory phenotype with high levels of perforin expression (46). With further identification of T cell responses, another group found higher frequencies of T effector memory and T follicular helper effector memory cells, but lower frequencies of T central memory, T follicular helper-central memory and T naïve cells in patients with severe COVID-19 compared to mild and moderate patients (47). Overall, patients with mild COVID-19 can provide robust antiviral immune responses, with CD8+ T cells expressing higher levels of cytotoxic molecules. However, the dysregulation of lymphoid functions, including Th1, Tregs, naïve and memory T cells, observed in severed COVID-19 patients all contribute to the increase in severe inflammatory conditions leading to hospitalizations (48). Another contributing characteristic of severe disease is the increase of exhaustion markers on T cells, indicted by the expression of inhibitory immune checkpoints and reduced expression levels of genes encoding cytokines and cytolytic molecules (49). While CD8+ T cells are important for targeting and killing virus-infected host cells, CD4+ T cells are responsible for stimulating inflammatory cytokines that recruit immune cells and are crucial for priming both CD8+ T cells and B cells (17, 50). Thus, enhancing T cell responses may provide greater protection against COVID-19. Effective antibody responses are stronger when T helper follicular cells and Th2 cells aid in the development of antibodies. Neutralizing antibodies, however, are short-lived, so understanding CD4+ and CD8+ T cell functions in protection are important as they develop long-lasting immunity to the pathogen.

While studies aim to distinguish lymphoid responses that discriminate between mild, moderate to severe COVID-19, it is critical to also determine the extent to which cells containing memory phenotypes exist after vaccination or infection. A recent study found that eight months after COVID-19 infection, immune responses pertaining to antibody production, memory B cell, CD4+ T cell, and CD8+ T cell memory all exhibited distinct kinetics (51). In this study, almost all individuals were positive for SARS-CoV-2 Spike and RBD IgG at 5-8 months post infection. Notably, they found spike specific memory B cells with no apparent half-life through, but found memory T cells with half-lives observed at 6 months (51, 52). Although these findings are not direct conclusions concerning protective immunity, the data sheds light to the durability of SARS-CoV-2 memory lymphocyte dynamics after 8 months post infection.

### Autoantibodies and Autoimmunity

Viruses are known as major environmental factors that trigger autoimmune disorders in genetically susceptible individuals. Viruses can trigger autoimmunity through a variety of mechanisms including molecular mimicry, bystander activation, and epitope spreading (53). Several studies focusing on COVID-19 rationalized that SARS-CoV-2 may also induce autoimmunity. One particular study found that COVID-19 patients exhibited a marked increase in autoantibody reactivities as compared to uninfected individuals, and also showed a high prevalence of autoantibodies against immunomodulatory proteins (including cytokines, chemokines, complement components and cell-surface proteins) (54). Another study found that individuals with life-threatening COVID-19 pneumonia developed neutralizing immunoglobulin G (IgG) autoantibodies against IFN-ω, IFN-α, or both- neutralizing the ability of the corresponding type I IFNs to block SARS-CoV-2 infection (55).

## Animal Models

While no animal model perfectly recapitulates the COVID-19 disease seen in humans, it is imperative to identify animal models for potential interventions, such as testing candidate vaccines and therapeutics, and investigate host-pathogen interactions. The use of different animal models allows investigators to study various aspects of infection and protection in COVID-19. As of now, nonhuman primates, rodents, ferrets, hamsters, and domesticated animals have all been established to model COVID-19.

### Mice

For over a hundred years, mouse *(Mus musculus)* models have been especially useful to study some aspects of human physiology or disease due to their ability to share mammalian features with humans and suffer from many of the same diseases. While a critical impediment to mouse models is the lack of appropriate human receptors, the use of selective breeding and genetic engineering has become very useful for following the progression of the disease. With respect to COVID-19, SARS-CoV-2 does not use mouse ACE2 as its receptor, making wild-type mice less susceptible to infection. To this end, there are several strategies that have been developed to surpass this problem. One approach is the generation of transgenic mice expressing human ACE2 in genetically modified mice (56–58). These transgenic mice express human ACE2 under the expression of a promoter, making them susceptible to SARS-CoV-2 infection; However, their differences in ACE2 expression results in a pathogenic range of mild to lethal disease. Another approach includes the adaptation of the virus to mouse ACE2 using reverse genetics. This was designed to remodel the interaction between SARS-CoV-2 spike protein and mouse ACE2 using a recombinant virus that can use mouse ACE2 for cell entry (6). Another adaptation method of SARS-CoV-2 to mice is the use of serial passing in the respiratory tract of aged mice (59). The mouse-adapted strain showed increased infectivity in mouse lungs and led to interstitial pneumonia and inflammatory responses. All of these methods are useful for the evaluation of therapeutics and vaccines for COVID-19.

### Ferrets

Following the fortuitous discovery of the natural susceptibility of ferrets (*Mustela putorius furo*) to human influenza viruses, ferrets became another popular animal model particularly for studying several other respiratory viruses, including respiratory syncytial virus, parainfluenza viruses, and SARS-CoV-1, as their lung physiology and disease manifestations recapitulate that of humans (60). Prior to the development of a COVID-19 model, ferrets were shown to contain critical SARS-CoV-2 binding residues in ACE2, further encouraging the use of a ferret model to study infection and transmission (61). A significant characteristic of using ferrets to study respiratory infections is their anatomic proportions of the upper and lower respiratory tract and the density of submucosal glands in the bronchial wall (60). Studies have identified a proper model of COVID-19 using ferrets as evidence suggests SARS-CoV-2 can be transmitted from individuals and are highly susceptible to infection. However, no animal model goes without its limitations, as ferrets were known to recapitulate only mild clinical symptoms and relatively low virus titers in the lungs of infected animals (62, 63).

### Syrian Hamsters

Syrian hamsters (*Mesocricetus auratus*) are small mammals that are widely used to study infections with respiratory viruses such as SARS-CoV-1, human metapneumovirus, human parainfluenza virus and influenza A virus. Alignment studies of ACE2 protein in humans, macaques, mice, and hamsters suggest that the spike protein of SARS-CoV-2 readily interacts with hamster ACE2 than murine ACE2, adding a pivotal role of using hamsters to study SARS-CoV-2 (64). Hamsters were reported to be a suitable experimental model for COVID-19, as hamsters infected with SARS-CoV-2 displayed apparent weight loss, developed severe pathological lesions in the lungs and showed efficient viral replication in the nasal mucosa and lower respiratory epithelial cells (64, 65). Because infection studies in hamsters are relatively completed quickly and are cost-effective, there is an increasing demand to use the model for possible screening of therapeutic agents. However, while hamsters are reported to develop moderate interstitial pneumonia leading to transient mild to moderate disease, the lack of research tools for this species remains low, adding a major caveat for the use of the model for the investigation of vaccines and therapeutics.

### Others

In efforts to determine alternative experimental models of COVID-19, the susceptibility of fruit bats, pigs, chickens, canine, and domestic cats to SARS-CoV-2 infection have also been studied. The significance of studying SARS-CoV-2 infection in other farmed and domesticated animals helps to not only characterize viral ecology but also provide insights into animal management for COVID-19 control. Due to the natural susceptibility of infection with coronaviruses, several studies have described implications of infection in fruit bats, however, because of the physiological differences in the immune system between humans and bats, bats are not considered suitable models for testing preventative or therapeutic measures (66). Rather, bats are suitable models to study potential reservoir hosts. Alternatively, SARS-CoV-2 has also been shown to replicate and transmit between domesticated cats (67). Upon SARS-CoV-2 challenge in cats, viral RNA in nasal, oropharyngeal and rectal swabs and bronchoalveolar lavage fluid was detected, however, all animals were clinically asymptomatic. While cats are not ideal infection models for testing vaccines and therapeutics, they serve to better understand the clinical course of SARS-CoV-2 in naturally susceptible host species and for risk assessment (67). Owing to their close contact with humans, studies focusing on environmental contamination and transmission efficiency using cat models might be critical for veterinary and public health authorities about the risk of cats as intermediate hosts. To this end, dogs have also been used to study SARS-CoV-2 infection and transmission. Studies found that dogs remained asymptomatic, have relatively low susceptibility to the virus and did not support viral replication (68, 69). Lastly, in efforts to determine viral infection in farmed animals, pigs, ducks, and chickens were not susceptible to SARS-CoV-2 infection (66, 68).

### Nonhuman Primates

Nonhuman primates are extensively used for biomedical research as they best recapitulate the pathogenesis of the human disease. Vaccine and therapeutic studies completed in both Old World and New World monkey species aim to treat and prevent viral infections. Indeed, no group of primates other than the anthropoid apes is more closely related to humans than are the Old World monkeys (70). SARS-CoV-2 infection studies have most commonly used Old World monkeys including, rhesus macaques (*Macaca mulatta*), cynomolgus macaques (*Macaca fascicularis*), African Green monkeys (*Chlorocebus sabaeus*), and baboons (*Papio* sp.), each developing different disease progression (19, 71, 72). Several other studies have also investigated SARS-CoV-2 infection in New World monkeys such as marmosets (*Macaca fascicularis*), however, these studies showed accelerated viral clearance and conveyed mild infection, making them less ideal for studying immune responses and testing vaccines and therapeutics (19, 73).

As one of the most commonly used nonhuman primates for biomedical research, rhesus macaques were shown to best recapitulate moderate disease as observed in the majority of human COVID-19 cases. After using a combination of multiple routes of inoculation with SARS-CoV-2 isolate nCoV-WA1–2020, radiographs from adult rhesus macaques showed pulmonary infiltrates starting one day post-infection with mild pulmonary infiltration primarily in the lower lobes. Clinical symptoms of infected macaques, therefore, showed transient, moderate disease (74). Another study similarly showed that SARS-CoV-2 could replicate and shed throughout the respiratory tract of rhesus macaques, including the oropharyngeal, nasal cavity, and alveoli. The authors of this study discussed the possibility of transmission between hosts was due to viral replication and shedding in the respiratory tract, while pulmonary infiltrates and histopathological lesions may be primarily due to the on-site replication in the lower respiratory tract (75). The examination of early events in SARS-CoV-2 infection revealed that infected animals developed clinical signs of viral infection (19). This was supported by viral-induced anemia and pulmonary dysfunction, as indicated by increased C-reactive protein, and decreased serum albumin and hemoglobin, and progressively increasing total serum CO2 levels. At necropsy, these animals also revealed findings of interstitial and alveolar pneumonia, with lungs being the most affected organ (19). However, these experiments did not investigate age as a factor considering COVID-19 disproportionally affects the elder population. To this end, a couple of studies compared young (3-5 years old) and old (15-year-old) rhesus macaques and aimed to determine age related differences in disease progression after SARS-CoV-2 inoculation (19, 76). To further elucidate age as a factor, these studies both found that SARS-CoV-2 caused more severe interstitial pneumonia in old macaques than in young macaques. Additionally, older macaques generated substantially reduced amounts of SARS-CoV-2-specific antibodies compared to young macaques (19, 76). Because of the striking differences seen between young and old macaques, rhesus macaques are exquisite animal models which make them useful for testing therapies and vaccines for elderly humans.

Additionally, studies aimed to determine protection against SARS-CoV-2 upon reinfection in rhesus macaques have also been established (77, 78). These studies are beneficial for elucidating the protective mechanisms against SARS-CoV-2 regarding immunological roles and critical for vaccine strategies, epidemiologic modeling, and public health approaches. After 4-5 weeks, animals that underwent primary infection and successfully recovered were rechallenged with SARS-CoV-2. Both studies found that after the primary infection there were induced humoral and cellular immune responses which provided protection against SARS-CoV-2 reinfection (77, 78). Indeed, rhesus macaques have shown to be suitable models for determining protective immune responses after a primary infection- however, additional studies that have a longer interval between the primary challenge and the rechallenge are needed to determine the extent of protection against SARS-CoV-2.

The cynomolgus macaque is another common Old-World monkey extensively used for biomedical research. In efforts to determine alternative animal models for COVID-19, several studies have established the cynomolgus macaque as a suitable model to investigate SARS-CoV-2 infection. One study compared SARS-CoV-2 infection with SARS-CoV and MERS-CoV infection, and found that after 4 days post-infection, macaques showed no overt clinical signs of disease, but did shed virus in nasal and throat swabs. These results best recapitulate human studies showing that asymptomatic individuals also shed virus. This study also presented data showing alveolar and bronchiolar epithelial necrosis, alveolar edema, hyaline membrane formation and accumulation of immune cells (79). A few other studies also compared the susceptibility to SARS-CoV-2 infection between different monkey species, including rhesus macaques and cynomolgus macaques and found both showed severe histopathological changes in lung, such as pneumonia, and inflammation in the spleen and liver. However, while both species had induced production of virus-specific antibodies and transient lymphopenia, showed high expression of inflammatory cytokines, and severe gross lesions on the lungs, spleen and lymph nodes, chest radiographs showed that pulmonary abnormalities were more server in rhesus macaques than in cynomolgus macaques (73). These data suggest that rhesus macaques are more susceptible to SARS-CoV-2 infection, and may ultimately be better models to study SARS-CoV-2 infection than cynomolgus macaques, as it mostly recapitulates human-like conditions.

Because African Green monkeys were found to best support the highest viral replication of SARS-CoV, several studies have also established this species as an animal model to investigate COVID-19 pathogenesis (72, 80–82). After inoculation of SARS-CoV-2, African Green monkeys were shown to develop mild, moderate or severe pulmonary lesions and pronounced viral pneumonia (72, 81). Similarly, another study showed that aged African Green monkeys (16 years old) developed ARDS and cytokine elevations similar to that reported in humans with severe COVID-19, however, the study did not have a direct comparison to younger monkeys (83). Additionally, to determine whether African Green monkeys display protection against secondary SARS-CoV-2 infection, monkeys were rechallenged after 5 weeks after the initial infection. The authors found no infectious virus in nasal samples, mucosal swabs nor BAL fluid after re-infection. These data suggest that African Green monkeys were protected from re-infection following the SARS-CoV-2 back-challenge (72). To this end, African Green monkeys have been shown to be suitable animal models to study COVID-19 pathogenesis and the host response to SARS-CoV-2 infection.

Baboons are commonly used as a model to study chronic diseases, such as obesity, heart disease, hypertension and osteoporosis. Because most COVID-19 cases were found in patients with pre-existing comorbidities, baboons may aid in the understanding of comorbidities and COVID-19 disease progression (84). In a study that compared multiple NHPs, baboons were shown to exhibit moderate to severe pathology, greater inflammation in lungs, higher viral titers, and significantly higher chest X-ray scores compared to rhesus macaques and marmosets (19). This study also demonstrated that age was a more corresponding attribute in baboons than in macaques, as they developed more severe inflammatory lesions. Therefore, the use of this model for understanding age- and comorbidity-related characteristics of COVID-19 may be useful.

It is known that no animal model can perfectly recapitulate disease progression as seen in humans; however, because of the controlled factors implicated in animal models, investigators are able to determine immunological mechanisms and pathologies that are otherwise nearly impossible to determine in humans.

## Vaccine Development

Effective therapeutic options for viral infections are mostly directed by blocking viral entry or replication or promoting durable cellular and humoral immunity for the uninfected population *via* vaccination. While researchers race to release a potent vaccination against COVID-19, the first priority is to determine safety and efficacy, as well as striving for long-term immunity. COVID-19 vaccines represent new classes of vaccine products, which show to be highly effective in preventing severe COVID-19 infections with hospitalization or death. The main platforms for vaccine development for COVID-19 are inactivated and live attenuated virus, non-replicating and replicating viral vector, nucleic acid-based, recombinant subunit vaccines, and more recently, the BCG vaccine (**Table 1**). As of July 2021, there are 108 vaccines in clinical development, and 184 vaccines in preclinical development (85). The current vaccines entering clinical trials are: 38 protein subunit, 17 non-replicating viral vectors, two replicating viral vectors, 11 DNA-based, 18 RNA-based, 16 inactivated virus, five virus-like particle, two live attenuated, two replicating viral vectors + antigen-presenting cells, and one non-replicating viral vector + plus antigen-presenting cell. The current COVID-19 vaccines that have been Authorized for Emergency Use are the Pfizer-BioNTech COVID-19 vaccine and Moderna COVID-19 vaccine (7).

**Table 1:** The possible advantages and limitations of the major vaccine platforms used for COVID-19 currently under clinical trials.

*Vaccine Platform*	Advantages	Limitations	Developer/Manufacturer
***Live Attenuated Virus (LAV)***	Intrinsic ability to stimulate immune responses that involve both innate and adaptive immunity. Allows viral entrance to host cell, as well as replication. A single dose is often enough to stimulate the immune response.	The virus can recover virulence and cause disease. Extensive testing regarding safety and efficacy. Cannot be given to immunocompromised people.	o Mehmet Ali Aydinlar University/Acıbadem Labmed Health Services A.S. o Codagenix/Serum Institute of India o Indian Immunologicals Ltd/Griffith University o Meissa Vaccines, Inc.
***Inactivated Virus***	Safer compared to LAVs, as it does not cause disease. Can be given to immunocompromised people.	Immunogenicity is weaker than LAVs. Requires multiple boosters in order to maintain immunogenicity.	o Sinovac o Wuhan Institute of Biological Products/Sinopharm o Beijing Institute of Biological Products/Sinopharm o Bharat Biotech o Institute of Medical Biology + Chinese Academy of Medical Sciences o Research Institute for Biological Safety Problems, Rep of Kazakhstan o Shenzhen Kangtai Biological Products Co., Ltd. o Valneva, National Institute for Health Research, United Kingdom o Erciyes University, Turkey o Shifa Pharmed Industrial Co o The Government Pharmaceutical Organization (GPO); PATH; Dynavax o Organization of Defensive Innovation and Research o Kocak Farma, Turkey o The Scientific and Technological Research Council of Turkey (TÜBITAK) o KM Biologics Co., Ltd. o Laboratorio Avi-Mex
***Viral Vectors***	High efficacy in gene transduction. Highly specific in gene delivery to specific host cells.	Immune responses against vectors are possible. Possible integration into the host genome.	o University of Oxford/AstraZeneca o CanSino o Biological Inc./Beijing Institute of Biotechnology o Gamaleya Research Institute o Janssen Pharmaceutical Companies o ReiThera/LEUKOCARE/Univercells o Institute Pasteur/Themis/Univ. of Pittsburg CVR/Merck Sharp & Dohme o Vaxart o University of Munich (Ludwig-Maximilians) o Merck & Co. + Themis + Sharp & Dohme + Institute Pasteur + University of Pittsburgh o University of Hong Kong, Xiamen University and Beijing Wantai Biological Pharmacy o Shenzhen Geno-Immune Medical Institute o City of Hope Medical Center + National Cancer Institute o Israel Institute for Biological Research o Aivita Biomedical, Inc. National Institute of Health Research and Development, Ministry of Health Republic of Indonesia o Bharat Biotech International Limited o Gritstone Oncology o Institute of Vaccines and Medical Biologicals, Vietnam o Tetherex Pharmaceuticals Corporation o German Center for Infection Research o CyanVac LLC
***Protein Subunit***	No live viral particles, thus is much safer and causes fewer side-effects. Can be given to immunocompromised people.	Long-term immunity is not definite. Multiple doses are needed.	o Novavax o Anhui Zhifei Longcom Biopharmaceutical/Institute of Microbiology o Chinese Academy of Sciences o Kentucky Bioprocessing, Inc o Sanofi Pasteur/GSK o Clover Biopharmaceuticals Inc./GSK/Dynavax o Vaxine Pty Ltd./CinnaGen Co. o Medigen Vaccine Biologics + Dynavax + National Institute of Allergy and Infectious Diseases (NIAID) o Instituto Finlay de Vacunas o Federal Budgetary Research Institution State Research Center of Virology and Biotechnology “Vector” o West China Hospital + Sichuan University o University Hospital Tuebingen o Vaxxinity o Center for Genetic Engineering and Biotechnology (CIGB) o Biological E. Limited o Nanogen Pharmaceutical Biotechnology o Shionogi o University Medical Center Groningen + Akston Biosciences Inc. o University of Saskatchewan o The University of Queensland o Walter Reed Army Institute of Research (WRAIR) o POP Biotechnologies and EuBiologics Co.,Ltd o Guangdong Provincial Center for Disease Control and Prevention/Gaozhou Center for Disease Control and Prevention o National Vaccine and Serum Institute, China o OSE Immunotherapeutics o USSF/Vaxform o Bagheiat-allah University of Medical Sciences o Baiya Phytopharm Co., Ltd. o Clover Biopharmaceuticals AUS Pty Ltd o Shanghai Zerun Biotechnology + Walvax Biotechnology + CEPI o Laboratorios Hipra, S.A.
***Nucleic Acid based***	Does not require handling infectious viral particles. DNA is temperature stable. mRNA is translated in cytosol, which prevents the integration of foreign genetic material into the host genome.	Immune responses are not as potent. DNA insertion may cause abnormalities to the host genome. RNA is not temperature stable. Limited protein immunogens.	o Inovio Pharmaceuticals/International Vaccine Institute o Osaka University/AnGes/Takara Bio o Cadila Healthcare Limited o Genexine Consortium o Moderna/NIAID o BioNTech/Fosun Pharma/Pfizer o Curevac o Arcturus/Duke-NUS o AnGes + Takara Bio + Osaka University o Zydus Cadila o Genexine Consortium o Arcturus Therapeutics o Imperial College London o Academy of Military Science (AMS), Walvax Biotechnology and Suzhou Abogen Biosciences o Entos Pharmaceuticals Inc. o Providence Health & Services o Chulalongkorn University o Symvivo Corporation o GeneOne Life Science, Inc. o University of Sydney, Bionet Co., Ltd Technovalia o Takis + Rottapharm Biotech o Providence Therapeutics o GlaxoSmithKline o Sanofi Pasteur and Translate Bio o Daiichi Sankyo Co., Ltd. o SENAI CIMATEC o ModernaTX, Inc. o Elixirgen Therapeutics, Inc o Shanghai East Hospital and Stemirna Therapeutics o MRC/UVRI and LSHTM Uganda Research Unit o AnGes, Inc
***BCG***	Stimulates trained immunity. Elicits protection amongst many pathogens, not limited to mycobacteria pathogens. Long term immunity	Minimal evidence for protection against COVID-19 May not prevent infection	o UMC Utrecht (BCG-CORONA) o Murdoch Children’s Research Institute (BRACE)

It is natural that viruses, notably RNA viruses, mutate after every replication, thus the best way to target a viral infection through vaccines is to target a component that is essential to its fitness, for example targeting S protein, which is critical for virus cell entry. With new variants rapidly spreading across the world, the main concern is that vaccines may not postulate protection against infection and disease. To further conquer protection against mutations, multivalent vaccines and live-attenuated vaccines may elicit stronger protection. However, these vaccine platforms come with their own safety concerns. A study demonstrated a RBD-Fc-based COVID-19 vaccine provided protection against SARS-CoV-2 and its mutants, SARS-CoV and SARS-rCoV (86). This vaccine, therefore, may have the potential to be a broad-spectrum vaccine to prevent infection of the emerging coronavirus. Additionally, like other RNA viruses, SARS-CoV-2 is prone to genetic evolution, lending itself the exquisite adaptations needed to survive and escape host immune responses. One of these adaptations include escaping immunological responses induced by vaccinations. Indeed, one large concern regarding the efficacy of currently distributed vaccines is how efficacious they are to providing protection against infection from new variants. A study demonstrated that while vaccines did not completed prevent infection by the COVID-19 delta variant, mRNA based vaccines were more efficacious at proving protecting against moderate and severe infection (87).

Children and pregnant persons are undoubtedly more susceptible to infection than other. It is currently advised by the CDC to vaccinate everyone 12 years and older, however, studies on vaccinating pregnant persons are limited (88). Although there is no clear explanation to disease severity outcome and pregnancy, several studies have observed severe illness and adverse birth outcomes among hospitalized pregnant persons with COVID-19 (89, 90). Thus, COVID-19 vaccines may reduce the risks of adverse pregnancy outcomes and disease severity, however, clinical trials testing the safety and efficacy of vaccines failed to include pregnant persons, thus much about vaccine administration remains unclear. However, a case study found SARS-CoV-2 IgG antibodies in the newborn of a pregnant person whom received a single dose of mRNA vaccine being first to show detectable antibodies in newborns (91). Following this study, others found pregnant and lactating persons generated robust humoral immunity with immunogenicity and reactogenicity similar to that observed in nonpregnant persons, providing evidence for the safety and efficacy of the mRNA COVID-19 vaccine (92, 93).

## Conclusion

SARS-CoV-2 is not the first coronavirus to cause mayhem in the human population, as seen in the past two decades. Rather, COVID-19 has been the greatest large-scale pandemic caused by a CoV yet, with over 203 million cases and approximately 4.3 million deaths, as of August 2021. Our efforts pertaining to the development of an effective vaccine and therapeutic against COVID-19 have been simultaneously challenged by the virus as it is continuously mutating. According to a US government SARS-CoV-2 Interagency Group (SIG), there are currently four variants of concern in circulation in the United States (US) including: B.1.1.7 (Alpha), B.1.351 (Beta), B.1.617.2 (Delta), and P.1 (Gamma). Of these variants, the Delta variant is more infectious and has led to increased transmutability, making it the predominant strain in the US. Ongoing data have suggested that the newly distributed vaccines are still effective against the new variants. According to the Centers of Disease Control and Prevention (CDC), vaccines continue to reduce an individual’s risk of contracting COVID-19, however, while these individuals may still fall ill (termed breakthrough infections), the vaccine provides them strong protection against serious illness and death (94). Additionally, it is now evident that dysregulated immune responses are the primary factor for disease outcome, providing insights in the development of effective therapeutics.

While this may not be the last CoV to emerge and cross the species barrier, research for better CoV detection and enhanced cross-reactivity may help prevent future CoV pandemics. It is clear that innate and adaptive immune responses are crucial for CoV infections, considering that the imbalance between a healthy immune response and a dysfunctional one can lay out the foundation for either successful viral clearance or disease progression. While extensive published and ongoing research have helped develop a better understanding of where we stand in the pandemic, we still face many challenges associated with COVID-19. With many efforts and updated guidelines by the CDC and WHO, we have developed better surveillance systems and case detections to reduce disease severity as the pandemic continued. However, in addition to respiratory tract related symptoms such as shortness of breath and cough, we have yet to completely understand SARS-CoV-2 infection as patients have also developed neurological complications (95). Patients with severe COVID-19 were shown to have neurologic manifestations such as acute cerebrovascular disease, impaired consciousness, and skeletal muscle injury (96). The limited neurological research of COVID-19 is mostly due to the inaccessibility of the human brain tissues. However, to overcome this challenge, researchers have developed an *in vitro* model using human stem cell-derived brain organoids to study the cellular and molecular effects of neurological SARS-CoV-2 infection (97). In this study, the researchers found that glia, cells essential for brain function and choroid plexus cells, cells that cover the capillary loops responsible for production of cerebrospinal fluid (CSF), were targeted by SARS-CoV-2, suggesting a novel mechanism by which SARS-CoV-2 induces neurological complications.

During the span of one year, researchers across the world have contributed significantly to the understanding of COVID-19, expanding from the development of therapeutics to vaccines. From the moment SARS-CoV-2 was known to the world to now, huge advancements have been made in efforts to understand its pathogenesis, disease outcomes, and immune responses, all of which have contributed to minimizing casualties as much as possible. The silver lining to the pandemic is the rapid development of the first mRNA-based vaccines, which have already been distributed across the globe and administered to about a billion human beings.

In the face of a pandemic, it is essential for researchers and health care advisors to thoroughly communicate with the public to successfully eliminate the viral threat and public dismay. COVID-19 will most likely not be the last global health emergency, efforts to minimize casualties in future pandemics through preparedness will allow us to deal with the next one in a more manageable manner. Through these efforts, it is important to never forget previous panics and turn to more pressing concerns; rather, we must learn to prepare for the next pandemic by building on previous pandemic progress. The COVID-19 pandemic affected many lives, and while it may have not been possible to completely avoid, it makes financial and economic sense to invest in preparedness for future health emergencies so that we are not catastrophically affected again.

## Author Contributions

RE and DS wrote the draft. RE prepared the illustrations. DK provided critical insights and supervision. All authors contributed to the article and approved the submitted version.

## Funding

U42OD010442 (NIH Office of the Director), P51OD011133 (NIH Office of the Director), NIH award # R01AI123780, R01AI134236 to DK and COVID-19 research grant from San Antonio Medical Foundation to DS.

## Conflict of Interest

The authors declare that the research was conducted in the absence of any commercial or financial relationships that could be construed as a potential conflict of interest.

## Publisher’s Note

All claims expressed in this article are solely those of the authors and do not necessarily represent those of their affiliated organizations, or those of the publisher, the editors and the reviewers. Any product that may be evaluated in this article, or claim that may be made by its manufacturer, is not guaranteed or endorsed by the publisher.
